# Knocking down claudin receptors leads to a decrease in prostate cancer cell migration, cell growth, cell viability and clonogenic cell survival

**DOI:** 10.1186/s43556-021-00053-0

**Published:** 2021-10-10

**Authors:** Qiang Liu, Hongliang Shen, Andrew Naguib, Robert M. Weiss, Darryl T. Martin

**Affiliations:** 1 Department of Urology, Yale University School of Medicine, New Haven, CT USA; 2 Department of Urology, Renji Hospital Affiliated To Shanghai Jiao Tong University School of Medicine, Shanghai, China; 3 Department of Urology, Beijing Friendship Hospital, Capital Medical University, Beijing, China

**Keywords:** Cell receptors, Molecular targeted therapy, Cancer cell survival, Cancer cell migration, Claudin-3 (Cldn3), Claudin-4 (Cldn4)

## Abstract

Prostate cancer is the most common solid organ malignancy in the United States, and has the highest probability of all cancers in becoming invasive. New molecular targets are needed to define and impede the growth and progression of advanced prostate cancers. Claudins (Cldns) are transmembrane proteins that regulate paracellular permeability and cell polarity, and their levels are elevated in many human cancers such as breast, ovarian, pancreatic, and prostatic cancers. Previously, we found that Cldn3 and Cldn4 are expressed in aggressive high-grade human prostate cancer specimens. We and others have shown that there are higher levels of Cldn3 and Cldn4 in metastatic human prostate cancer cells than in normal human prostate cells. The result of targeting Cldn3 and Cldn4 expression on the growth and viability of prostate cancer cells has not been elucidated. Human prostate cancer PC3 and LNCaP cells were transfected with Cldn3 or -4 small interfering RNAs (siRNAs). Cldn3/Cldn4 siRNA treatment resulted in a greater than 85% decrease in the protein levels of Cldn3 and Cldn4, which was accompanied by a 30–40% decrease in prostate cancer cell growth and a 60–65% reduction in cell viability. There was decreased cell migration with Cldn3 and Cldn4 siRNA in both PC3 and LNCaP cells and a 60–75% decrease in the number of clones when treated with siCldn3 or siCldn4 compared to control. Knocking down Cldn3/Cldn4 affects prostate cancer cell growth and survival and may have therapeutic implications.

## Introduction

Prostate cancer is the most common solid organ malignancy in the US. There is an estimated 248,530 new prostate cancer cases and over 34,000 deaths expected for 2021 [1]. Of all cancers that occur in men, prostate cancer has the highest probability of becoming invasive when considering all age groups [1]. Currently, there are no curative treatment options for late-stage prostate cancer, and death becomes inevitable. Thus, new treatment targets need to be identified to eliminate therapeutic failure and to improve prostate cancer patient outcomes.

Tight junction proteins are directly involved in the barrier and adhesive function between adjacent cells and tight junctions aid in the regulation in paracellular permeability and cell polarity [2–7]. Abnormal tight junction proteins and function as well as aberrant cell polarity are noted in cancer cells [8–12]. Additionally, tight junction proteins also are deemed to be implicated in tumorigenesis and metastasis. Claudins are transmembrane proteins that belong to a family of tight junction proteins. Claudins have four transmembrane domains, including two short cytoplasmatic domains and two extracellular loops [13]. In examining the amino acid composition of the extracellular domains there is much variety between the claudin isoforms. Claudins play a role in embryogenesis and also are found to be altered in many human cancers [7]. Specifically, overexpression of Cldn3 and Cldn4 tight junction proteins promote tumorigenesis in breast, endometrial, gastric, kidney, ovarian, and uterine cancers [11]. In addition, Cldn4 is overexpressed in primary and metastatic prostate cancer, and Cldn3 is strongly expressed in the majority of prostate cancers [14, 15]. Previously, we showed, using human prostate cancer biopsy specimens, that higher levels of CLDN3 and CLDN4 expression are found in intermediate and high-risk prostate cancers compared to low and very low risk prostate cancer specimens [Martin DT, Lee JS, Liu Q, Galiana G, Sprenkle PC, Humphrey PA et al.: Targeting prostate cancer with *clostridium perfringens enterotoxin* functionalized nanoparticles co-encapsulating imaging cargo enhances magnetic resonance imaging specificity, *Submitted*]. Also, we and others have shown that claudin-3 (Cldn3) and claudin-4 (Cldn4) are overexpressed in prostate cancer cells compared to normal prostate cells [14, 16, 17] [Martin DT, Lee JS, Liu Q, Galiana G, Sprenkle PC, Humphrey PA et al.: Targeting prostate cancer with *clostridium perfringens enterotoxin* functionalized nanoparticles co-encapsulating imaging cargo enhances magnetic resonance imaging specificity, *Submitted*].

Targeting Cldn expression has been shown to be effective in inhibiting tumor growth in several tumor models. For example, silencing Cldn3 using small interfering RNA (siRNA) resulted in suppression of ovarian xenograft tumor growth and metastasis [18]. Similarly, blocking Cldn4 using a monoclonal antibody in ovarian and pancreatic xenograft mouse models inhibited tumor growth [19]. Furthermore, exposing *Clostridium perfringens enterotoxin* (CPE), a potent cytolytic toxin, to its natural receptors Cldn3 and Cldn4 has been shown to induce CPE-mediated cytotoxicity in prostate cancer cells and ovarian tumors [17, 20, 21].

We hypothesize that Cldn3 or Cldn4 may be potential therapeutic targets for the management of prostate cancer. In this investigation, we examined Cldn expression in human prostate cancer cells. Although Cldn3 and Cldn4 have been shown to be expressed in prostate cancers, and was the focus of CPE intervention, targeting Cldn3 and Cldn4 expression in relation to prostate cancer cell survival rates has not been investigated. Herein, we target Cldn3 and Cldn4 expression in prostate cancer cells using Cldn3 and Cldn4 siRNAs and assess its impact on cell growth, migration, viability, and clonogenic survival.

## Results

### Claudin expression in human prostate cancer and normal prostate cells

**Fig. 1:**
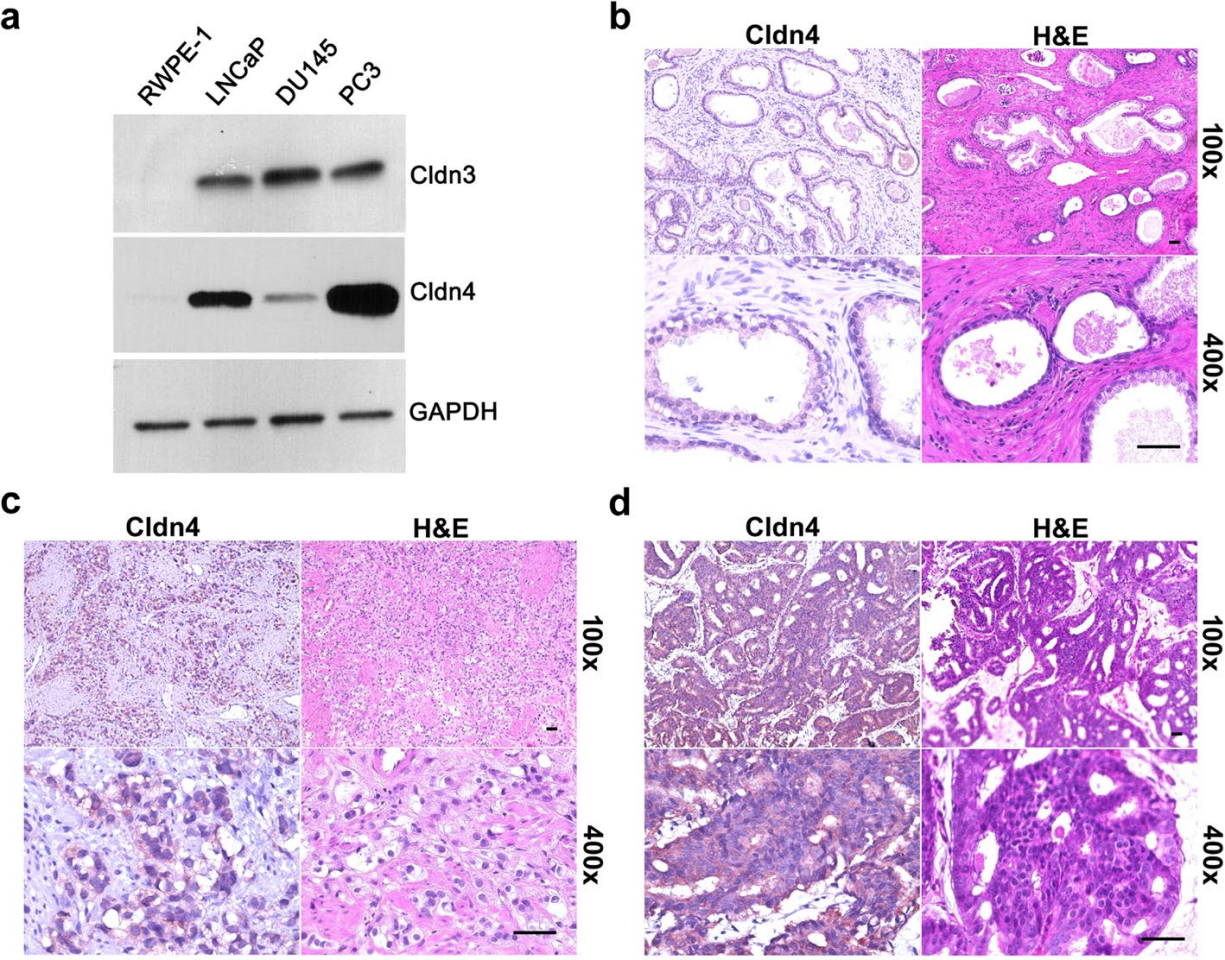
Cldn expression in human prostate cells and tissue specimens. **a** The levels of Cldn3 and Cldn4 were higher in metastatic human prostate cancer (PC3, LNCaP, and DU145) cells compared to benign prostate (RWPE-1) cells. Immunohistochemistry was performed on (**b**) human benign prostatic hyperplasia specimen, (**c**) human high-grade prostate cancer specimen 1 (Gleason grade group 4 +), and (**d**) human high-grade prostate cancer specimen 2 (Gleason grade group 4 +) for Cldn4 (brown). Representative H&E and IHC staining are shown at 100× and 400×﻿. Scale bars = 50 μM

Western blots show that Cldn3 and Cldn4 levels are higher in metastatic prostate cancer cells (PC3, LNCaP, and DU145) compared to normal prostate cells (RWPE-1) (Fig. 1a). In addition, we confirmed that Cldn4 is expressed in higher grade prostate cancer specimens compared to benign prostatic hyperplasia specimens (Fig. 1b-d).

### Knocking down Cldn3 and Cldn4 in in vitro human prostate cancer cells

**Fig. 2:**
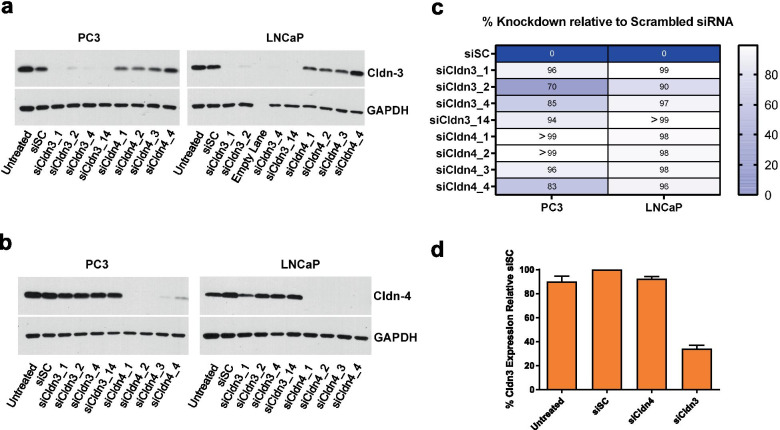
Knock down of Cldn3 and Cldn4 in human prostate cancer cells. Western blots showing the effects of Cldn3 siRNAs (siCldn3) and Cldn4 siRNAs (siCldn4) on the expression of (**a**) Cldn3 and (**b**) Cldn4 respectively, in human PC3 and LNCaP prostate cancer cells after 72 h. Four Cldn3 and Cldn4 siRNAs were tested. **c** A heatmap demonstrates the percent knockdown (i.e., Color scale is used where white represents a 100% knockdown of protein expression and dark blue represents a 0% knockdown of protein expression) for each siRNA target sequence for PC3 and LNCaP prostate cancer cells. **d** Relative Cldn3 protein levels in LNCaP prostate cancer cells after 2 weeks of siCldn3 and siCldn4 treatment

Four siRNAs for Cldn3 (siCldn 3_1, siCldn 3_2, siCldn 3_4, siCldn 3_14) and for Cldn4 (siCldn 4_1, siCldn 4_2, siCldn 4_3, siCldn 4_4) were examined for their efficacy in knocking down Cldn3 and Cldn4 levels, respectively. All efficiently knocked down their respective targeted Cldn levels (Fig. 2a, 2b). In examining the protein levels of Cldn3 and Cldn4 after 72 h, we generated a knocked down of greater than or equal to 70% for both PC3 and LNCaP human prostate cancer cells when treated with siCldn3s and siCldn4s (Fig. 2c). Specifically, we generated a Cldn3 knockdown of 85% and 97% for PC3 and LNCaP human prostate cancer cells, respectively, when treated with siCldn3_4 and a Cldn4 knockdown of > 99% and 98% for PC3 and LNCaP human prostate cancer cells, respectively, when treated with siCldn4_1. The percent knockdown by these siRNAs was made in comparison to siSC. Based on the knockdown of Cldn3 and Cldn4 using PC3 and LNCaP prostate cancer cells, siCldn3_4 and siCldn4_1 were selected for all subsequent functional studies. In addition, we treated LNCaP prostate cancer cells with siCldn3 and noted a 70% knockdown of Cldn3 expression after 2 weeks (Fig. 2d).

### Cell growth is affected by Cldn3 and Cldn4 knockdown

**Fig. 3:**
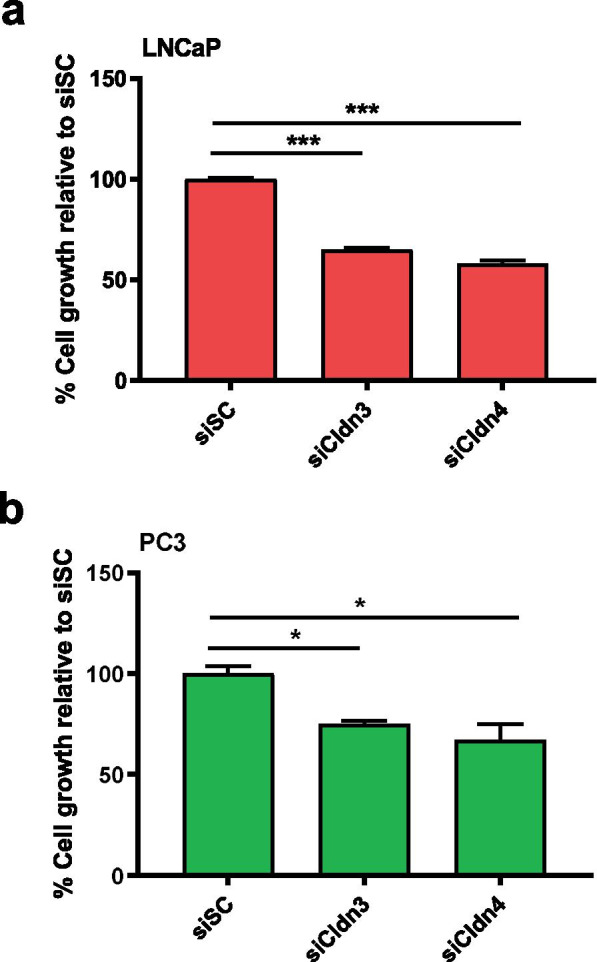
Assessment of cell growth upon siCldn treatment. Cell growth of (**a**) LNCaP and (**b**) PC3 human prostate cancer cells following siCldn3 or siCldn4 treatment relative to growth following siSC. Data are shown as mean ± SD of 3 to 4 independent experiments. * represents *p* < 0.05 and *** represents *p* < 0.001

Knocking down Cldn3 and Cldn4 expression appears to be androgen independent as both human prostate cancer cell lines PC3 (androgen independent) and LNCaP (androgen dependent) had similar growth outcomes. Prostate cancer cell growth was decreased by 35% (*p* < 0.001) and by 42% (*p* < 0.001) for LNCaP cells (Fig. 3a) and by 25% (*p* < 0.05) and by 33% (*p* < 0.05) for PC3 cells (Fig. 3b), upon treatment with siCldn3 and siCldn4, respectively, compared to siSC.

### Cell viability is decreased upon siCldn3 and siCldn4 knockdown

**Fig. 4:**
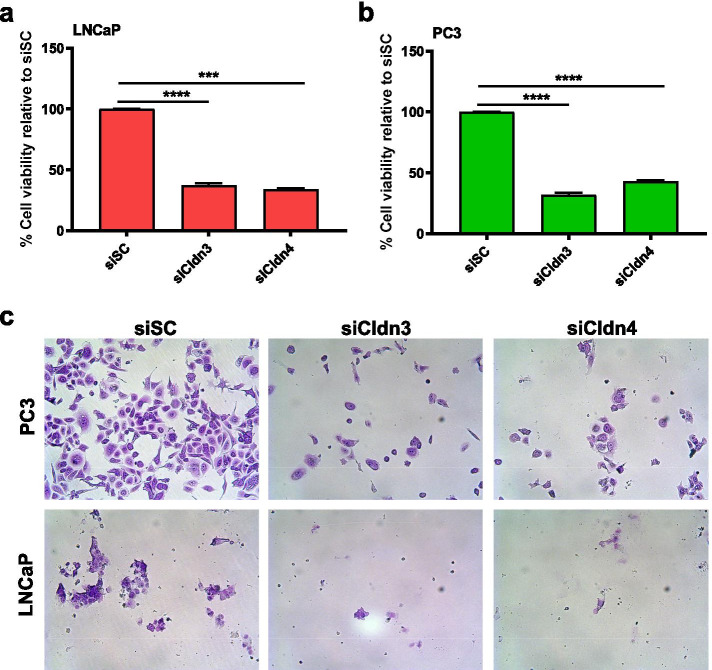
The effects of knocking down Cldn3 and Cldn4 on cell viability. Cell viability was assessed on human (**a**) LNCaP or (**b**) PC3 prostate cancer cells following siCldn3 or siCldn4 treatment. **c** Crystal violet staining was performed to visualize viable cells. Data are shown as mean ± SD of 3 to 4 independent experiments. *** represents *p* < 0.001 and **** represents *p* < 0.0001

Cytotoxicity WST colorimetric assays were performed on LNCaP and PC3 prostate cancer cells exposed to 100 nmol/L of siCldn3 or siCldn4. There was a 63% (*p* < 0.0001) and 66% (*p* < 0.001) decrease in viability for LNCaP cells (Fig. 4a), and a 68% (*p* < 0.0001) and 57% (*p* < 0.0001) decrease in viability for PC3 cells (Fig. 4b), when treated with siCldn3 or siCldn4, respectively. A crystal violet assay was used to assess prostate cancer cell viability (Fig. 4c).

### Clonogenic ability was decreased in prostate cancer cells upon treatment with Cldn3 and Cldn4 siRNA

**Fig. 5:**
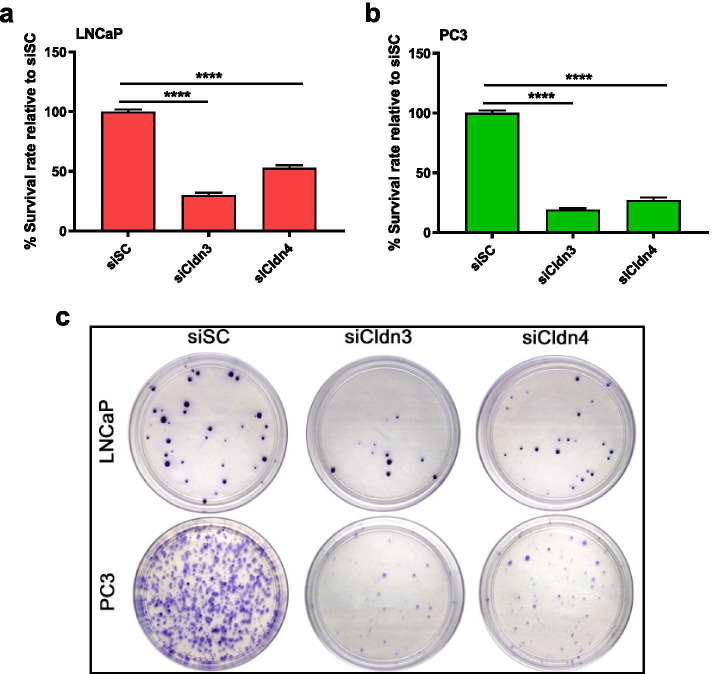
Clonogenic survival upon siCldn treatment. Treatment with siCldn3 and siCldn4 was performed on human prostate cancer (**a**) LNCaP and (**b**) PC3 cells. **c** Crystal violet staining was performed to visualize colony formation. Data are shown as mean ± SD of 3 to 4 independent experiments where **** represents *p* < 0.0001

Knock down of Cldn3 or Cldn4 in LNCaP and PC3 cells significantly decreased long-term clonogenic growth. The clonogenic survival rates decreased by 70% (*p* < 0.0001) and 47% (*p* < 0.0001) for LNCaP colonies (Fig. 5a), and by 81% (*p* < 0.0001) and 73% (*p* < 0.0001) for PC3 colonies (Fig. 5b) when treated with siCldn3 or siCldn4, respectively. Crystal violet staining was performed to visualize the prostate cancer colonies after siCldn3 and siCldn4 treatment (Fig. 5c).

### Cell migration decreased after treatment with Cldn3 and Cldn4 siRNA

**Fig. 6:**
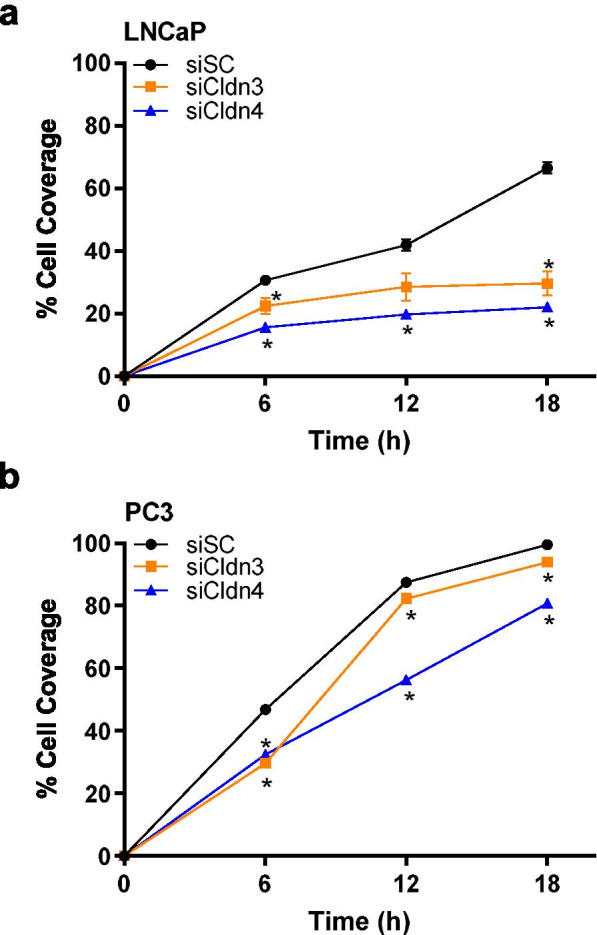
Cell migration of prostate cancer cells treated with siCldn. The percent difference of (**a**) LNCaP and (**b**) PC3 prostate cancer cell scratch coverage between treatments (siCldn3, orange line and siCldn4, blue line) and control (siSC, black line) was determined at 6, 12, and 18 h. Data are shown as mean ± SD of 3 to 4 independent experiments where * represents *p* < 0.05

Cell migration was evaluated in LNCaP and PC3 cells by determining the percent cells covering the scratch after siCldn3 or siCldn4 treatment. We showed a difference in percent scratch coverage at 6 h (8%, 15%), at 12 h (13%, 22%), and at 18 h (37%, 45%) compared to the siSC control in LNCaP cells (Fig. 6a) when treated with siCldn3 or siCldn4, respectively. In PC3 cells the difference in percent cell coverage at 6 h was (17%, 14%), at 12 h (5%, 31%) and at 18 h (6%, 19%) compared to the siSC control when treated with siCldn3 or siCldn4, respectively (Fig. 6b). Both siCldn3 and siCldn4 had a significant inhibitory effect on cell migration of prostate cancer cells.

## Discussion

The expression of Cldn3 and Cldn4 is tissue specific and has been described to be up-regulated in ovarian, breast, and prostate cancers [14, 16, 22–24]. Martin et al. found higher levels of CLDN3 and CLDN4 expression in higher-risk human prostate cancer specimens compared to lower risk cancer specimens [Martin DT, Lee JS, Liu Q, Galiana G, Sprenkle PC, Humphrey PA et al.: Targeting prostate cancer with *clostridium perfringens enterotoxin* functionalized nanoparticles co-encapsulating imaging cargo enhances magnetic resonance imaging specificity, *Submitted*]. The similar expression patterns of Cldn3 and Cldn4 in prostate cancers may suggest a coordinated regulation and raises the possibility for an effective targeted treatment strategy [25]. Furthermore, although the function of Cldn3 and Cldn4 is usually associated with tight junctions in normal tissues, they do not appear to have this role in tumors, suggesting a conceivable role of Cldn3 and Cldn4 in cancer progression [7, 26].

The goal of our study was to examine the differences in Cldn3 and Cldn4 protein levels according to growth characteristics of prostate cancer cells. We demonstrated an in vitro tumor-specific claudin effect. We showed that decreasing Cldn expression resulted in a less aggressive cell type characterized by a decrease in cell growth, cell migration, clonogenic cell survival, and cell viability. Similar to our findings in prostate cancer, higher levels of Cldn3 and Cldn4 expression have been observed in aggressive ovarian cancers, than in normal ovarian cells [21]. Huang and colleagues showed that knocking down CLDN3, using siCldn3, reduced tumor growth in a mouse ovarian cancer model [18].

Although, the above reports are consistent with a correlation of elevated Cldn expression levels with increased tumor aggressiveness, this correlation has not been universal and to some extent appears to be tumor specific. In contrast to the findings of Huang et al. showing that knocking down CLDN3 reduced ovarian tumor growth, Shang et al. demonstrated that knocking down Cldn3 and Cldn4 expression in an ovarian cancer xenograft model led to an increase in tumor growth and metastatic potential [18, 27]. They also demonstrated that knocking down CLDN3 and CLDN4 increased migration and invasion in vitro, and suggested that the deficit of CLDN3 and CLDN4 expression is associated with poor prognosis [27].

In accord with these findings in ovarian carcinoma, Cldn4 expression has been reported to be reduced in the majority of gastric cancers and lower expression levels correlate with poorly-differentiated gastric adenocarcinomas [28]. In addition, the overexpression of Cldn4 in gastric carcinoma was correlated with improved patient prognosis and overexpression inhibited gastric carcinoma cell migration and invasion, although it did not affect cell growth [29]. Also, the loss of Cldn4 expression in a colorectal cancer patient was associated with end stage disease [30].

Herein, we showed that knocking down Cldn3 or Cldn4 expression in prostate cancer cells decreased cell survival. This led us to examining if knocking down of Cldn3 and Cldn4 simultaneously using both siCldn3 and siCldn4 would lead to a synergistic effect. We found that the combination of Cldn3 and Cldn4 knockdown did not provide any further cell survival advantage compared to single treatments (data not shown). These data suggest that there is some overlap or coordinated regulation in Cldn3 and Cldn4 function in prostate cancer cell growth and migration.

Our present study demonstrates that targeting Cldn3 and Cldn4 may be therapeutically useful for managing prostate tumors that have high Cldn3 or Cldn4 expression levels. Furthermore, these receptors may be useful in enhancing prostate cancer detection.

## Materials and Methods

### Cells/Tissues

PC3 (CRL-1435), LNCaP (CRL-1740), and DU145 (HTB-81) prostate cancer cells were acquired from the American Type Culture Collection (ATCC). PC3, LNCaP, and DU145 cells were maintained in F12-K, RPMI-1640, and Eagles Minimum Essential medium, respectively. All prostate cancer cells were supplemented with 10% fetal bovine serum and 1% glutamine. RWPE-1 (CRL-11609) benign prostate cells, immortalized by human papillomavirus 18, were obtained directly from the ATCC and maintained in Keratinocyte Serum Free Medium (Invitrogen) and augmented with 0.05 mg/ml BPE and 5 ng/ml EGF. All cells were maintained as previously described [31]. In addition, all participating patients provided informed consent and were offered enrollment into a specimen repository approved by the Institutional Review Board at Yale University.

### siRNA treatment

Human prostate cancer LNCaP and PC3 cells which were transfected with claudin-3 siRNA (siCldn3), claudin-4 siRNA (siCldn4), or scrambled control siRNA (siSC) were used as previously described [31]. The siCldn3 target sequence (siCldn3_4) was: 5’-GGUCGGCCAACACCAUUAU -3’ (sense) and the siCldn4 target sequence (siCldn4_1) was: 5’- GGCUACAGGUAAUGGGCAU -3’ (sense). We used a negative control, scrambled/nonsense siRNA (siSC) sequence: 5’-AACGUACGCGGAAUACUUCGA-3’ (Dharmacon), which demonstrated the specificity of our Cldn siRNA sequences. After 72 h, the prostate cancer cells were evaluated for cell growth, viability, migration, and for clonogenic survival. In addition, protein expression was assessed at 72 h and 2 weeks.

### Western blotting

Western blot was performed as previously described [31]. Briefly, a radioimmunoprecipitation assay (Cell Signaling Technology) enhanced with cOmplete™ protease inhibitor cocktail (Roche Applied Science), 1 mM sodium fluoride, 2 μg/ml aprotinin, and 1 mM phenylmethylsulfonyl fluoride was used to lyse the prostate cells. Quantification of protein was assessed by a Bradford assay. Primary antibodies, such as Cldn3 and Cldn4 (Novus), and secondary antibodies, such as anti-mouse and anti-rabbit (Cell Signaling Technology), were used. Protein signal was detected using Chemiluminescence (Thermo Scientific, Rockford IL).

### Cell growth assay

Prostate cancer cells were treated with siCldn3, siCldn4, or siSC. They were plated in a 6-well plate at a density of 1.5 × 10^4^ cells per well. After 72 h, cells were stained with 0.4% trypan blue (Gibco, Life Technology), and then using a TC10 cell counter (Bio-Rad), were counted.

### Cytotoxicity assay

Human prostate cancer cells were plated in a 96-well plate at a density of 5000 cells per well. They were treated with siCldn3, siCldn4, or siSC. After 72 h, cell viability was measured (at 450 nm) using a WST tetrazolium reagent with the appropriate controls including a background control. The assay was performed following the manufacturer’s instructions (Clontech Laboratories, CA). Origin Lab Data Analysis Software was used to measure the % inhibition of cell viability compared to control (siSC).

### Crystal violet staining

Human prostate cancer cells were treated with siCldn3, siCldn4, or siSC. A 96 well plate was used and 5000 cells were plated per well. After 48 h, cells were fixed with ice cold methanol before being stained with 0.5% crystal violet dye [32]. Prostate cancer cells were washed with deionized water. Plates were then dried and photographed.

### Clonogenic survival

PC3 and LNCaP human prostate cancer cells were treated with siCldn3 or siCldn4 for 72 h and then plated at 18 viable cells per cm^2^. Colonies were formed after 3 weeks and then fixed with 4% paraformaldehyde. At this point, colonies were stained with crystal violet dye and washed with deionized water. Cell clumps were considered colonies if they were larger than or equal to fifty cells. Colonies were imaged and counted [33]. The effectiveness of the employed agents on cell survival was determined by using the survival rate calculation as previously described [31].

### Migration assay

LNCaP and PC3 human prostate cancer cells were treated with siCldn3, siCldn4, or siSC (scrambled control) for 48 h. Cells were seeded in triplicate in culture medium containing 1% FBS in a 24-well plate. Cell confluency was reached after 24 h. A scratch was made in the confluent monolayer of prostate cancer cells. Cells that migrated into the scratch area were counted and quantified at 6, 12, and 18 h [33].

### Statistics

Student’s t-test and repeated-measures ANOVA analysis were used as part of the statistical analysis. All statistical tests were two-sided. Statistical significance was achieved at *p* < 0.05. Statistical analysis was carried out using GraphPad Prism 8.0. Results are presented as mean + / − SD in which values of significance are shown as *, *p* < 0.05; **, *p* < 0.01; ***, *p* < 0.001; and ****, *p* < 0.0001 unless otherwise indicated.

## Data Availability

Listed in manuscript.

## Abbreviations

ATCC: American Type Culture Collection
BPH: Benign Prostatic Hyperplasia
Cldn3: Claudin-3
Cldn4: Claudin-4
CPE: *Clostridium perfringens enterotoxin*
siCldn3: Claudin-3 siRNA
siCldn4: Claudin-4 siRNA
siRNA: Small interfering RNA
siSC: Scrambled siRNA
